# Can archived lesion smears be used to identify *Leishmania* species in regions with high species diversity?

**DOI:** 10.1590/S1678-9946202567039

**Published:** 2025-06-27

**Authors:** João Guilherme Pontes Lima Assy, Nara Karyne Delduck Feitosa, Jaqueline Alves Delprete, Vanessa Kehdy, Rose Grace Brito Marques, Expedito José de Albuquerque Luna, José Angelo Lauletta Lindoso, Lucia Maria Almeida Braz

**Affiliations:** 1 Núcleo Técnico de Vigilância em Saúde, Santarém, Pará, Brazil; 2 Instituto de Infectologia Emilio Ribas, São Paulo, São Paulo, Brazil; 3 Universidade de São Paulo, Faculdade de Medicina, Departamento de Medicina Preventiva, São Paulo, São Paulo, Brazil; 4 Universidade de São Paulo, Faculdade de Medicina, Instituto de Medicina Tropical de São Paulo, São Paulo, São Paulo, Brazil

**Keywords:** Archived lesion smears, *Leishmania* identification, Amazonian region

## Abstract

The identification of *Leishmania* species is crucial for eco-epidemiological purposes and may be useful for clinical management. Notably, archived smear slides can be valuable in this scenario. ITS-1 PCR followed by sequencing was used to identify *Leishmania* species from archived lesion smears of patients with suspected cutaneous leishmaniasis in Santarem city, Para State, Brazil. A total of 44 microscopically positive lesion smears were analyzed, of which 34 yielded positive PCR results. Of these, 22 were subjected to Sanger sequencing and 15 were successfully sequenced, revealing five *Leishmania* species. This study demonstrates the applicability of molecular testing on archived samples. ITS-1 sequencing effectively differentiated between species, revealing significant diversity of *Leishmania* in the Brazilian Amazon.

## INTRODUCTION

Cutaneous leishmaniasis (CL) is an infectious disease caused by the protozoan *Leishmania.* The correct identification of *Leishmania* species is important for epidemiological purposes and for prescribing the appropriate treatment^1^. Parasitological tests are used to diagnose the disease, but they cannot identify the species. However, it is possible to differentiate *Leishmania* species using the polymerase chain reaction (PCR), which can be performed on different biological samples, such as smears stained on slides and previously examined by microscopy^2-5^. The internal transcribed spacer-1 (ITS-1) region of ribosomal RNA, a target for PCR, contains numerous polymorphisms suitable for species-level differentiation, along with conserved regions of diagnostic value^6^. This is an extremely sensitive target and, after sequencing, capable of identifying *Leishmania* species^7^. The literature indicates that Giemsa-stained smears and tissues embedded in paraffin are potential clinical specimens for confirming the diagnosis of leishmaniasis and identifying *Leishmania* species circulating in a given area^2-5^. However, few studies have identified *Leishmania* species from archived slide samples in Brazil.

Such types of samples are particularly practical for describing species distribution in Brazil, especially in resource-limited rural settings, and they facilitate epidemiological, ecological, clinical, and pharmacological studies. We aimed to identify *Leishmania* species from lesion smears archived on slides from patients with suspected cutaneous leishmaniasis in the Central Amazonia, Brazil.

## MATERIAL AND METHODS

This project was approved by the Ethics Committee of the Hospital das Clinicas, Faculdade de Medicina da Universidade de Sao Paulo (CAAE Nº 60871822.5.0000.0068).

In total, 54 lesion smears were obtained from 54 patients with suspected cutaneous leishmaniasis (CL) attending an outpatient CL reference center in the city of Santarem, Para State, Brazil, from 2019 to 2022. The patients lived in rural areas or worked in the forests of the Lower Amazon, engaging in activities such as mining, logging, agriculture, and livestock farming. The 54 slides were the only ones maintained in the Nucleo Tecnico de Vigilancia em Saude (NTVS) laboratory after microscopy examination (1000x). Fifty-four skin lesion imprint slides were prepared, Giemsa-stained, and analyzed by microscopy. After two to four years, the archived Giemsa-stained smears were scraped from the slides into microtubes containing 200 µL of distilled water, and DNA was subsequently extracted using the QIAamp DNA Mini Kit (Qiagen).

The 54 extracted DNA samples were subjected to PCR targeting the ITS1 region. The ITS1 locus was amplified using the primers LITSR (5’-CTG GAT CAT TTT CCG ATG-3’) and L5.8S (5’-TGA TAC CAC TTA TCG CAC TT-3’), yielding products of 310-330 bp, according to the assay conditions described by Schonian *et al*.^8^ and modified by Godoy *et al*.^6^. The 25 µL PCR reaction mixture consisted of: 1X reaction buffer, 4.0 mM MgCl_2_, 0.2 mM dNTPs, 0.4 µM LITSR and L5.8S primers, 2 U Taq polymerase, and 100 ng of extracted DNA. PCR was performed using a Nexus GSX1 Mastercycler Thermocycler (Eppendorf, Germany) with the following cycling parameters: initial denaturation at 94 °C for 6 min, followed by 35 cycles of denaturation at 94 °C for 20 s, annealing at 53 °C for 30 s, and extension at 72 °C for 60 s, with a final extension at 72 °C for 1 min.

For PCR product analysis, 6 µL aliquots of each sample were electrophoresed on a 2% agarose gel (Argagen, Spain) at 80 V for one hour. Depending on the experiment, the molecular weight markers were either a 100 bp or 50 bp ladder (Invitrogen Life Technologies, USA) or a 100 bp ladder (Sinapse Inc., USA). Gels were stained with ethidium bromide, and DNA bands were visualized under ultraviolet light.

Positive PCR products underwent Sanger sequencing to determine the nucleotide base sequence of the ITS1 locus, which enabled the differentiation of *Leishmania* species by comparing the obtained sequences with well-characterized species sequences from GenBank. Prior to sequencing, the PCR fragments were purified using the GeneClean II kit (MP Biomedicals, USA) according to the manufacturer's instructions. Sequencing was performed at the Human Genome and Stem Cell Research Center of the Instituto de Biociencias, Universidade de Sao Paulo, using the oligonucleotides LITSR and L5.8S, following the established protocol. Sanger sequences were analyzed using BioEdit software and their similarity to existing sequences was evaluated using Basic Local Alignment Search Tool (BLAST). Accession codes were obtained following the deposition of the sequences for each species in NCBI (National Center for Biotechnology Information).

## RESULTS

**Figure 1:**
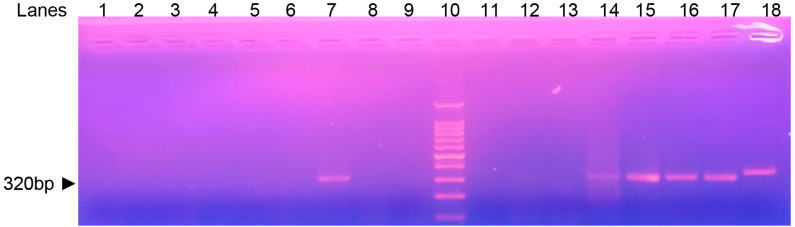
Agarose gel electrophoresis showing representative *Leishmania* spp. amplicons obtained by ITS-1 PCR, using DNA samples extracted from Giemsa-stained slides of patients with suspected cutaneous leishmaniasis from Santarem city, Para State, Brazil. Lanes 14-17: positive samples; Lanes 1-6, 8-9, 11-13: negative samples; Lane 10: 100 bp DNA molecular weight ladder; Lane 7: *L. (V.) braziliensis* positive control; Lane 18: *L. (L.) amazonensis* positive control.

A total of 54 lesion smears from 54 patients were examined, of which 81.5% (44/54) tested positive for cutaneous leishmaniasis (CL) by microscopy (gold standard). Among these microscopy-positive samples, 34 were also positive by PCR-ITS-1, whereas none of the microscopy-negative smears tested positive by PCR-ITS-1. This resulted in a sensitivity of 77.3% (34/44) and a specificity of 100% (10/10). Figure 1 shows positive and negative samples and controls.

Sequencing analysis was successful for 22 of the 34 amplified samples; 12 samples did not meet the required conditions.

Subsequently, 64.7% (22/34) of the amplified samples (ITS1-PCR) underwent Sanger sequencing, followed by analysis using BLASTn software. Table 1 shows the five *Leishmania* species identified in this analysis: *L.* (*V.*) *braziliensis* (3 samples), *L.* (*L*.) *amazonensis* (3 samples), *L.* (*V.*) *lainsoni* (3 samples), *L.* (*V.*) *guyanensis* (2 samples), and *L.* (*V.*) *naiffi* (1 sample). In three samples, only the *Viannia* subgenus could be identified, without determining the specific species [*L*. (*V*.) *braziliensis* and *L*. (*V*.) *guyanensis; L*. (*V*.) *guyanensis, L*. (*V*.) *shawi*, and *L*. (*V*.) *panamensis*; and *L*. (*V*.) *panamensis* and *L*. (*V*.) *guyanensis*]. Seven of the 22 Sanger sequences were not analyzed due to poor quality.

**Table 1:** Identification of *Leishmania* species in archived Giemsa-stained lesion smears from cutaneous leishmaniasis patients in Santarem city, Para State, Brazil, performed using ITS1-PCR sequencing and BLAST analysis.

*Leishmania* species (Accession codes)	Percent identity (%)	Lenght of the sequence	Query cover (%)	e-value
*L. (V.) braziliensis* (PV478026)	100	297	100	8e-152
*L. (V.) braziliensis (PV478028)*	99.32	297	99	6e-148
*L. (V.) braziliensis (PV483775)*	98.04	204	100	1e-93
*L. (L.) amazonensis* (PV478037)	100	313	100	1e-160
*L. (L.) amazonensis (PV478068)*	99.69	322	100	5e-164
*L. (L.) amazonensis (PV478067)*	99.69	324	100	4e-165
*L. (V.) lainsoni* (PV478025)	99.66	301	99	1e-150
*L. (V.) lainsoni (PV478029)*	99.65	289	100	4e-145
*L. (V.) lainsoni (PV478035)*	99.64	274	100	2e-137
*L. (V.) guyanensis* (PV478036)	100	267	100	1e-134
*L. (V.) guyanensis (PV483780)*	100	198	100	5e-97
*L. (V.) naiffi* (PV483779)	98.54	203	100	3e-95
*L. (V.) panamensis/L. (V.) guyanensis*	100	195	100	2e-95
*L. (V.) panamensis/ L. (V.) guyanensis/L. (V.) shawi*	99.19	250	99	2e-121
*L. (V.) braziliensis /L. (V.) guyanensis*	100	169	100	6e-81

## DISCUSSION

Our study demonstrated that it is feasible to extract DNA and perform PCR on Giemsa-stained slide smears with acceptable sensitivity. We achieved amplification in 77.3% (34/44) of the smears, which were similar results to those found by Al-Jawabreh *et al*.^3^ Almazan *et al*.^5^, who also used Giemsa stained smears and ITS1 as the PCR target. In the study by Al-Jawabreh *et al*.^3^ ITS1-PCR showed a sensitivity of 87% and a specificity of 100% compared to microscopy. In Argentina, Almazan *et al*.^5^ identified *Leishmania* belonging to the *Viannia* subgenus from DNA extracted from Giemsa-stained slides stored for 12 years, in which 74% (74/100) of the smears were amplified by ITS-1-PCR. This indicates that *Leishmania* DNA can be successfully detected from archived biological material. However, specifically in our study, the hot climate of Santarem, state of Para, Brazil, where slide smears were stored, may have resulted in low DNA quality and PCR interference from dye residues, potentially diminishing the molecular positivity rate (77.3%) and the quality of sequencing.

Several authors have also successfully detected *Leishmania* DNA using paraffin-embedded tissue extraction methods^4,9-11^. In Brazil, Lima *et al*.^10^ identified *Leishmania* by PCR followed by SSU rDNA sequencing in paraffin-embedded skin samples stored for more than 30 years. Of the 33 samples amplified, 26 were successfully sequenced: 16 were identified as *L.* (*L*.) *amazonensis*, and the remaining 10 were found to belong to the *Viannia* subgenus. In a study conducted in Iran, DNA extraction was performed on seven lymph node paraffin blocks collected from 1994 to 2007, confirming the presence of *Leishmania tropica* in positive cases^9^. Prestes *et al*.^4^ also demonstrated the feasibility of applying molecular techniques for diagnosing human parasites in paraffin-embedded tissues. Finally, *Leishmania major* was diagnosed in a dog with cutaneous manifestations via DNA extracted from a snout biopsy block^11^.

In Iran, Motazedian *et al*.^2^ performed kinetoplast DNA minicircle (kDNA) PCR on samples from stained slides, archived for four years, from suspected cases of CL. They observed PCR positivity in 89.13% (82/92) of the samples, compared to 82.6% (76/92) positivity by microscopy. This study highlighted the superior sensitivity of PCR over microscopy, a finding that contrasts with our results. Note that Motazedian *et al*.^2^ used kDNA as the PCR target, whereas our study employed ITS1. Despite kDNA's higher sensitivity compared to ITS1, it shows low specificity. Furthermore, *Leishmania* species identification is possible via ITS-1 sequencing, but not kDNA sequencing. The ability of ITS1-PCR followed by sequencing to differentiate species of Old World leishmaniasis has been previously demonstrated. In southeastern Iran, ITS1-PCR amplified samples were sequenced and subjected to phylogenetic analysis, revealing the coexistence of two species in the province: *L. tropica* (88.5%) and *L. major* (11.5%)^12^. Sequence analysis of 18S RNA and ITS1 amplicons identified *L. major* in all samples from CL lesions in the Kurdistan, Iraq^13^. ITS1 is a well-known and widely used marker for identifying the *Leishmania* genus, particularly in Old World samples. In our study, ITS1-PCR followed by Sanger sequencing identified five species of *Leishmania*, demonstrating an important diversity of species circulating in the New World and the effectiveness of the ITS1 target in distinguishing them. Almeida *et al*.^14^ also identified nine different species of *Leishmania* after sequencing ITS-1 PCR amplicons from 76 samples in the Roraima State, in the Brazilian Amazon. Although ITS1 is a good marker for identifying the *Leishmania* genus in Old World samples and showed some success in our study and in that by Almeida *et al*.^14^, further research may be necessary to identify the most suitable marker for distinguishing species within the *Viannia* subgenus.

In Brazil, *L.* (*L*.) *braziliensis* is the primary causative agent of CL; however, six other *Leishmania* species have also been identified by the Ministry of Health as causing CL, particularly in the Amazon: *L.* (*V.*) *guyanensis, L.* (*V.*) *naiffi, L.* (*V.*) *shawi, L.* (*V.*) *lindenbergi, L.* (*V.*) *lainsoni*, and *L.* (*L.*) *amazonensis* ^14^- ^17^. More recently, Almeida 2021 suggested the introduction of two additional species from neighboring countries, namely *L.* (*V.*) *panamensis* and *L.* (*L.*) *mexicana*, in Roraima State, Northern Amazon^14^. Other studies in the Amazon have also documented high species diversity in CL cases. Coelho *et al*.^15^, using monoclonal antibodies and isoenzymes, identified *L.* (*V.*) *guyanensis* (73%, 153/209) as the most prevalent species in Manaus. *L.* (*V.*) *guyanensis* was also the predominant species in the Amapa State (84.2%, 32/38), detected via hsp70-PCR sequencing^17^. In contrast, Telles *et al.* ^16^ identified 65.6% of *L.* (*V*.) *braziliensis* in Acre State using hsp70-PCR-RFLP.

## CONCLUSION

Our study demonstrates that Giemsa-stained slide smears are a viable and effective method for sampling in PCR-based analyses. The results underscore the potential and value of applying molecular techniques to archived samples, enabling retrospective analyses and complementing molecular diagnostics. Additionally, our findings highlighted the wide range of *Leishmania* species circulating in Central Amazon indicating that *L.* (*V*.) *braziliensis* may not be the predominant species in this region.
